# Effectiveness of extracorporeal shockwave therapy in three major tendon diseases

**DOI:** 10.1007/s10195-015-0361-z

**Published:** 2015-07-02

**Authors:** Christian Carulli, Filippo Tonelli, Matteo Innocenti, Bonaventura Gambardella, Francesco Muncibì, Massimo Innocenti

**Affiliations:** Orthopaedic Clinic, University of Florence, Largo P. Palagi 1, 50139 Florence, Italy

**Keywords:** Extracorporeal shockwave, Calcific tendonitis of the shoulder, Achilles tendinopathy, Epicondylitis

## Abstract

**Background:**

Extracorporeal shockwave therapy is a conservative treatment for several painful musculoskeletal disorders. The aim of the study was the assessment of the relief from pain by the shockwave therapy in a population of consecutive patients affected by specific pathologies.

**Materials and methods:**

A group of consecutive patients were studied and treated. They were affected by calcific tendonitis of the shoulder (129 patients), chronic Achilles tendinopathy (102 patients), and lateral epicondylitis of the elbow (80 subjects). Each patient had 3 applications with a monthly interval, and was followed up at 1, 6, and 12 months after treatment. Results were evaluated by the numeric rating scale (NRS) in all cases, the Constant Murley Score for the assessment of the shoulder function, the American Orthopaedic Foot and Ankle Society Score for subjects affected by chronic Achilles tendinopathy, and the Oxford Elbow Score for those affected by a lateral epicondylitis of the elbow.

**Results:**

One year after treatment, the results were considered satisfactory with an almost complete resolution of symptoms. There were statistically significant results at the 12-month follow-ups regarding the mean NRS score (from 6.25 to 0.2), the Constant Murley Score (from 66.7 to 79.4), the Oxford Elbow Score (from 28 to 46), and the AOFAS (from 71 to 86).

**Conclusions:**

Extracorporeal shockwave therapy may be considered a safe, economic, and effective treatment for several chronic musculoskeletal disorders, allowing satisfactory pain relief and improvement of function ability.

**Level of evidence:**

Level IV.

## Introduction

Extracorporeal shock wave therapy (ESWT) is one of the great advances in orthopaedics over the last 20 years [1]. Initially indicated for the treatment of kidney stones [2], it has been applied in cases of bone non-unions, and then in several musculoskeletal disorders, given the satisfactory clinical outcomes reported in different randomized clinical trials and cohort studies. The main indications have been the following: lateral epicondylitis of the elbow, calcific tendonitis of the rotator cuff, plantar fasciitis, Achilles and patellar tendinopathy, and pubalgia [1, 3–13]. A reduction of pain and a good recovery of articular function have been obtained in most cases [13–17], even if in high-level athletes a more aggressive strategy is recommended to allow a quick return to sports activities [18].

The mechanism by which ESWT may produce a clinical effect is still uncertain. Several theories have been proposed: a mechanical effect by increasing the pressure in the calcium deposition causing fragmentation; a molecular effect with induction of an inflammatory response with neovascularization and then a chemotactic action and phagocytosis of calcific deposits; an analgesic effect by inhibiting the activation of the serotonergic system, and peripheral denervation. Probably, a combination of angiogenic and analgesic effects explains the overall outcomes on the target tissues [7, 8, 19–23]. Direct and indirect biologic effects of ESWT vary proportionally to the amount of energy and to the type of frequency applied; moreover, the shockwave driving tool influences the induced modifications on the target tissue [24].

The aim of the present retrospective study is the evaluation of the clinical outcomes in a population of patients affected by common musculoskeletal disorders treated by ESWT.

## Materials and methods

From January 2011 to March 2013, 311 consecutive patients were selected and treated by ESWT for specific painful musculoskeletal disorders at the authors’ institution. One-hundred and twenty-nine were affected by a calcific tendonitis of the shoulder, 102 by an Achilles tendinopathy, and 80 by a later epicondylitis of the elbow.

The mean age was 48.5 (range 19–80); 230 were male, and 81 female. Inclusion criteria were: adult patients with clinical and instrumental diagnosis of lateral epicondilytis of the elbow, chronic Achilles tendinopathy, and calcific tendonitis of the shoulder; persistent symptoms for at least 3 months; failure or partial resolution of symptoms after conservative (medical and physical) treatment; no recent history of trauma or chronic joint instability; no recent related surgery.

Exclusion criteria were: patients with a clinical but not instrumental diagnosis of any tendon disease; subjects who had not tried any conservative approach; subjects referring an inadequate duration of proper medical or physical treatments. The institutional review board allowed the retrospective analysis of patients’ data and outcomes. Demographic data of the selected patients are reported in Table 1.

**Table 1:** Demographics and characteristics of the patients

	Shoulder calcific tendonitis (*n* = 129)	Achilles tendinopathy (*n* = 102)	Elbow lateral epicondylitis (*n* = 80)
Male/female	92/37	46/56	45/35
Mean age (range)	47.5 (19–70)	48 (22–80)	50 (20–76)
Mean duration of symptoms (weeks)	4.3 (3–7)	6.7 (2–9)	3.9 (2–6)
Dominant side affected	72	64	56
Previous treatments (number of subjects)			
NSAIDs	31	24	19
Other analgesics	52	41	28
Physical therapy	21	16	12

Pain assessment in all patients was conducted before treatment by an 11-point numeric scale (numeric rating scale, NRS). The clinical evaluation was conducted by the Constant Score for the assessment of shoulder function [25]; the American Orthopaedic Foot and Ankle Society Score (AOFAS) [26] for subjects affected by chronic Achilles tendinopathy; and the Oxford Elbow Score [27] for those suffering a lateral epicondylitis of the elbow. All patients gave their consent to the treatment and follow-up.

A single device generating shockwaves (ReflecTron^®^, HMT, Switzerland) was used in all cases. The energy level and number of shots were adapted to the specific pathology according to the protocols supplied by the manufacturer. Each patient had 3 ESWT applications at monthly intervals. Each session consisted of 2400 shockwave applications with an intensity depending on the site and the pathology observed (Table 2). No local anaesthesia was given before the treatment. All patients were treated by two experienced orthopaedic surgeons.

**Table 2:** Active level of ESWT

*Disease*	Pulses and energy of ESWT
Calcific tendonitis of the shoulder	2400 pulses at >0.20 mJ/mm^2^
Achilles tendinopathy	2400 pulses at 0.08–0.33 mJ/mm^2^
Lateral epicondylitis of the elbow	2400 pulses at <0.12 mJ/mm^2^

All subjects were followed up at 1, 6, and 12 months after the last application. The clinical evaluation consisted of NRS and function evaluation by the administration of the above mentioned specific scores (Table 3). Particular attention has been focused on the use of analgesic drugs, reported complications after the ESWT applications, and the need for any further instrumental study.

**Table 3:** Clinical and functional scores

	Baseline	1 month	6 months	12 months
Numeric rating scale (NRS)				
Calcific tendonitis of the shoulder^a^	6.5 ± 1.4 (4–9)	5.9 ± 1.3 (3–9)* *p* = 00.013	1.2 ± 0.8 (0–3)* *p* < 0.001	0.2 ± 0.4 (0–1)* *p* < 0.001
Achilles tendinopathy^a^	6.9 ± 1.2 (5–9)	5.3 ± 1.1 (4–8)* *p* < 0.001	1.7 ± 0.8 (0–3)* *p* < 0.001	0.3 ± 0.5 (0–2)* *p* < 0.001
Lateral epicondylitis of the elbow^a^	6.6 ± 1.2 (4–9)	4.2 ± 1.0 (3–6)* *p* < 0.001	0.9 ± 0.8 (0–3)* *p* < 0.001	0.1 ± 0.3 (0–1)* *p* < 0.001
Functional scores				
Constant Murley Score	66.7 ± 4.3 (56–76)	73.7 ± 3.9 (59–78)* *p* = 00.012	78.3 ± 2.6 (64–80)* *p* < 0.001	79.4 ± 1.4 (70–80)* *p* < 0.001
AOFAS	71 ± 5.6 (63–80)	72 ± 3.2 (67–75)* *p* < 0.001	77 ± 2.4 (72–84)* *p* < 0.001	86 ± 1.9 (82–90)* *p* < 0.001
Oxford Elbow Score	28 ± 2.7 (23–35)	35 ± 2.5 (31–38)* *p* = 0.0016	42 ± 2.6 (36–47)* *p* < 0.001	46 ± 2.6 (42–50)* *p* < 0.001

* Paired Student t-test, compared to baseline (*p* < 0.05)

^a^The use of pain regulating drugs was reported by 34 patients (12.0 %), with a peak of utilization in the first 3 days, once daily

The statistical analysis was performed by a sample size calculation based on a priori assumption of *p* = 0.05. All data were tested for the normal distribution using the Kolmogorov–Smirnov test. The Student t-test was used to perform the analysis for the scores, testing each disease separately. For each parameter, three coupled samples were calculated (before treatment–1 month, before treatment–6 months, before treatment–12 months) (Table 3).

## Results

Two-hundred and eighty-three patients completed the follow-up period. Twenty-eight subjects were lost: none of them was lost due to conditions or complications related to the procedures.

**Fig. 1:**
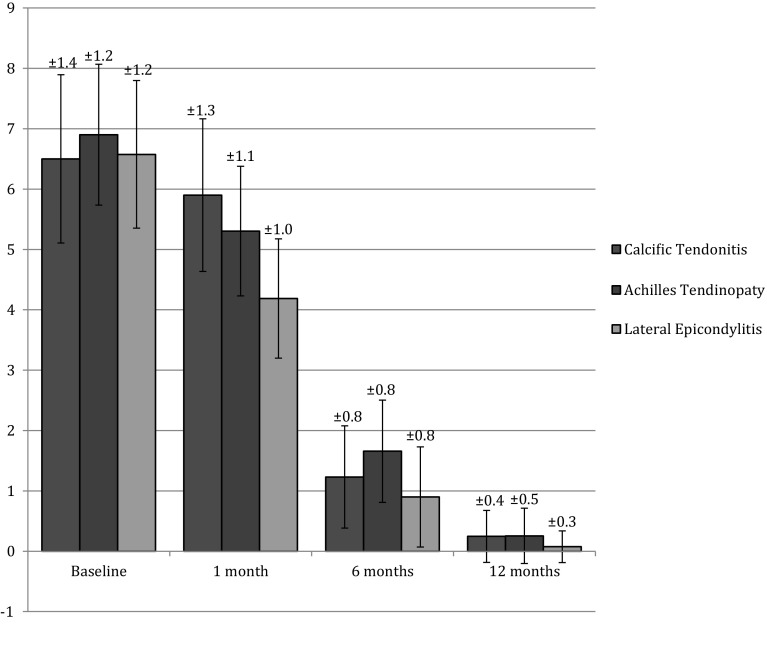
NRS scores after treatment

**Fig. 2:**
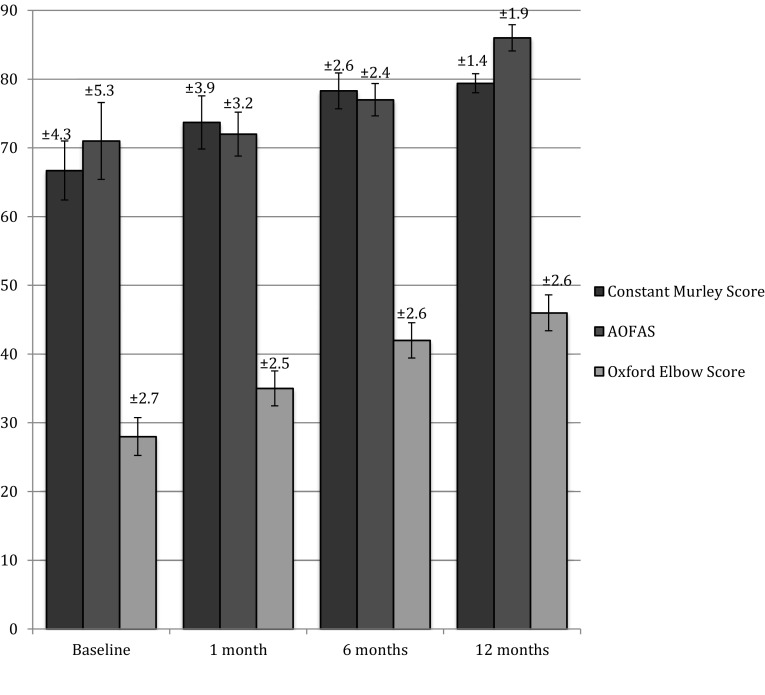
Constant Murley, AOFAS, and Oxford Elbow Scores after treatment

No complications were recorded. In 42 cases, the patients reported the presence of cutaneous bruises after the applications. The overall mean NRS score was 6.25 (range 4–9) before the treatment. One month after the first application, the mean NRS score was 4.9 (range 3–9), 1.2 at 6 months (range 0–3), and finally 0.2 at 12 months (range 0–2). Considering single pathologies, patients showed an improvement in any score: mean NRS, mean Constant Murley Score for shoulders, mean Oxford Elbow Score for elbows, and mean AOFAS Score for feet (Table 3). Over the months of follow-up we recorded a progressive maintenance of results (Figs. 1, 2). The use of pain regulating drugs was reported by 34 patients (12.0 %), with peak utilisation on the first 3 days, once daily. In 12 cases (4.2 %), the pain did not show a significant decrease so an ultrasound or MRI examination was necessary to understand the causes of the persistency of symptoms.

## Discussion

Shockwave therapy represents an innovative approach for the management of painful chronic musculoskeletal diseases, particularly in the case of failure of a previous conservative treatment. This treatment has to be considered safe, minimally invasive, versatile, and with low costs [28].

In the present study, as reported in the literature, after a latency of days to a few weeks after treatment, all patients reported a clinical benefit, with a significant decrease of pain, improvement in function, and a fair use of analgesics. Focusing attention on the specific pathologies, our outcomes are in line with the latest reports.

Lateral epicondylitis of the elbow was treated by ESWT in five recent RCTs, mostly of high quality [29–33]. In two of these, no significant differences were found up to 48 weeks after the treatment between ESWT and placebo [31, 32]. Spacca et al. [31] found significant differences between ESWT and placebo on pain (0.5 versus 6.5) and grip strength (46 versus 36) 12 weeks after the treatment. Pettrone et al. [29] found similar significant differences in pain at the 12-week follow-up. Collins et al. [33] found significant difference in pain during activity in favour of the ESWT group. There is conflicting evidence for the effectiveness of ESWT versus placebo in the short term and evidence of no difference in effect on the mid-term and long-term follow-up.

Several studies have confirmed the benefits of ESWT for the treatment of calcific tendonitis of the shoulder [24, 34, 35]. Particularly, it has been reported that high-energy ESWT (EFD ≥ 0.28 mJ/mm^2^) are more effective than low-energy doses (EFD < 0.28 mJ/mm^2^) in the improvement of the shoulder function and pain resolution. Gerdesmayer et al. [35] enrolled 144 patients with a randomized level of energy (high or low). Both types of ESWT resulted in a significant improvement at the 6-month evaluation, but high-energy ESWT induced a higher outcome on the Constant Murley Score. Calcific deposits disappeared in the same percentage of patients in both groups. Cacchio et al. [34] used a different score (University of California–Los Angeles UCLA Shoulder Rating Scale) to evaluate shoulder function after ESWT treatment versus placebo of calcific tendonitis of the shoulder. Significant differences in favour of ESWT versus placebo were reported at the 6-month follow-up.

ESWT is effective as a conservative approach in the treatment of chronic Achilles tendinopathy. This has been recently confirmed by some important RCTs [12, 36, 37]. Rasmussent et al. [12] showed improvements in the treatment with ESWT versus placebo at a 12-week follow-up. The mean AOFAS Score increased from 74 to 81 in the placebo group and from 70 to 88 in the ESWT group (*p* = 0.05). Better results were seen in the ESWT group at 8 and 12 weeks (*p* = 0.01 and *p* = 0.04, respectively). Rompe et al. [37] showed an improvement in the VISA-A score (specific for Achilles tendon pathologies) which increased in two groups: one with a treatment by eccentric loading exercises and one with eccentric loading + ESWT. The better outcomes were registered for the second group.

Despite the positive results, this study has some limitations. First of all, there was no patient randomization or use of placebo for any treatment. Moreover, there was no control group and the analysis of outcomes was performed without blind examiners. Our protocols were based on a 3-session ESWT application that, in our opinion, represents a reasonable approach, even if it is not the only approach. Finally, the diagnosis of each pathology was established by all authors, even if the ESWT applications were performed by two dedicated surgeons.

In conclusion, we believe that treatment with extracorporeal shockwaves may be a useful option in the management of several chronic musculoskeletal pathologies, particularly after the failure of a conventional approach. The wide spectrum of applications, the low related costs, and its safety represent the strength of this modern approach to the treatment of disabling musculoskeletal diseases.
